# CPM Signals for Satellite Navigation in the S and C Bands

**DOI:** 10.3390/s150613184

**Published:** 2015-06-05

**Authors:** Rui Xue, Yanbo Sun, Danfeng Zhao

**Affiliations:** College of Information & Communication Engineering, Harbin Engineering University, Harbin 150001, China; E-Mails: xuerui0216@hotmail.com (R.X.); zhaodanfeng@hrbeu.edu.cn (D.Z.)

**Keywords:** multi-band, combined navigation, modulation signal, CPM

## Abstract

Frequency allocations in the L band suitable for global navigation satellite system (GNSS) services are getting crowded and system providers face an ever tougher job when they try to bring in new signals and services while maintaining radio frequency compatibility. With the successive opening of the S and C bands to GNSS service, the multi-band combined navigation is predicted to become a key technology for future high-precision positioning navigation systems, and a single modulation scheme satisfying the requirements in each band is a promising solution for reducing user terminal complexity. A universal modulation scheme based on the continuous phase modulation (CPM) family suitable for the above bands’ demands is proposed. Moreover, this paper has put forward two specific CPM signals for the S and C bands, respectively. Then the proposed modulation schemes, together with existing candidates, are comprehensively evaluated. Simulation results show that the proposed CPM signals can not only satisfy the constraint condition of compatibility in different bands well and reduce user terminal complexity, but also provide superior performance in terms of tracking accuracy, multi-path mitigation and anti-jamming compared to other candidate modulation schemes.

## 1. Introduction

With the recent modernization of GPS and GLONASS signals and the emerging Compass and Galileo systems, the number of navigation satellite signals in space is increasing drastically and anticipated to surpass 400 by 2030. Such a large number of signals will further exacerbate an already crowded radio spectrum in the 1164–1610 MHz L band and negatively impact the performance of all navigation systems sharing these limited resources [1].

In order to solve the above problem, the International Telecommunication Union (ITU) has successively allocated the S band (2483.5–2500 MHz) and C band (5010–5030 MHz) to satellite navigation services. Although signal performance in a single S or C band cannot surpass the L band’s due to the smaller available bandwidth and the higher path losses [2,3,4], the signal combination of L band and S or C band could improve positioning accuracy and timing performance and comprehensively promote the performance of radio navigation services [3,5,6]. Therefore, the multi-band combined navigation and compatibility among different navigation systems has become a research hotspot in recent years [7,8].

Compared to multi-frequency signals in the L band, multi-band multi-frequency signals can not only mostly reduce ionosphere delays and strengthen ionospheric correction capability, but also ease carrier phase integer ambiguity resolution to improve robustness and position accuracy, and enhance the anti-interference ability of GNSS signals [2,3,5,6]. As is well-known, signal structure is one of the decisive factors for GNSS and modulation is one of the key technologies which must be resolved during the system design and upgrading process. CPM has been widely used in the field of satellite communication [9,10], which has high spectrum efficiency and constant-envelope features. Besides, it has many other excellent characteristics, such as a large number of alternative waveforms, flexible parameter adjusment, better compatibility with existing signals and so on.

In the L band, a special subclass of CPM with semi-integer modulation index h (h=H+1/2, H∈N) greater than one and satisfying a constraint $h/2T_{cpm}=n\;\times \;1.023\;\mathrm{MHz}$ can exhibit a similar spectral main lobe and yield comparable navigation performance compared to conventional binary offset carrier (BOC) denoted as BOC(*n*, *m*), where m × 1.023 MHz is the spread spectrum code rate, n × 1.023 MHz is the frequency of sub-carrier and $T_{cpm}$ denotes the CPM signal symbol time [11]. The IRNSS will transmit navigation signals in the lower S band. BPSK(*m*) and BOC(*n*, *m*) centered on a frequency close to 2491 MHz are the specific waveforms [12]. Meanwhile, minimum shift keying (MSK) as a potentially promising C band signal waveform that has been investigated for the Galileo system [13], which is a special case of CPM. Unfortunately, the above modulation schemes can’t meet the requirement of compatibility in the S and C bands very well due to relatively high side lobes. Furthermore, different modulation waveforms employed by each band undoubtedly increase the user terminal complexity in the multi-band combined navigation mode. In view of this, we propose a universal modulation scheme based on the CPM family and design two specific CPM signals as S and C band solutions by virtue of their properties, which will make a single modulation waveform design possible and accelerate the practicality of multi-band combined navigation technology.

The rest of this paper is organized as follows: Section 2 describes the mathematical model and power spectrum density (PSD) of CPM signals. The Section 3 provides a comprehensive evaluation criterion for GNSS signal design and introduces analytical methods in terms of anti-jamming performance. The proposed CPM signals together with other candidates are comprehensively evaluated in Section 4 and Section 5, respectively. Finally, we conclude the paper in Section 6.

## 2. CPM Signal

### 2.1. Mathematical Model

The time-domain representation of CPM signals can be expressed as [14,15]:

(1) $$s(t)=\sqrt{\frac{2E}{T}}\cos(2\pi f_{0}t+\phi (t,\text{α})+\varphi_{0})$$

where the E, T, $f_{0}$ and $\varphi_{0}$ are the symbol energy, symbol period, carrier frequency and initial phase respectively, and φ(t,α) is the information-carrying phase denoted as:

(2) $$\phi (t,\text{α})=2\pi h\sum_{i=-\infty }^{\infty }\alpha_{i}\int_{-\infty }^{t}g(\tau -iT)d\tau ,\;-\infty <t<\infty$$

where the *M*-ary data symbols $\alpha_{i}$ take values ±1, ±3, ⋯, ±(M−1), h is modulation index of k/p, normally the function g(t) is a smooth pulse shape over a finite time interval 0≤t≤LT and zero outside. Thus, by choosing different pulses g(t) and varying the parameters h and M, a great variety of CPM schemes can be obtained. For convenience, we use the notation LRC for a raised cosine pulse of length L symbol intervals. For example, 2RC is a raised cosine pulse of length 2T. Likewise, the rectangular pulse of length L is denoted as LREC.

**Figure 1:**
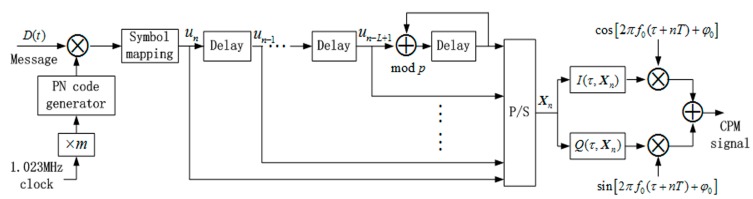
CPM signal generator scheme for GNSS.

The CPM can be decomposed into continuous phase encoder (CPE) and memoryless modulator (MM) [16], which greatly reduces implementation complexity. The signal generation block diagram of CPM as future GNSS modulation is shown in Figure 1, where $I(\tau ,X_{n})$ and $Q(\tau ,X_{n})$ are mapping functions of in-phase and quadrature branches, defined as:

(3) $$I(\tau ,{\text{X}}_{n})=\cos\bar{\phi }(\tau ,{\text{X}}_{n})$$

(4) $$Q(\tau ,{\text{X}}_{n})=\sin\bar{\phi }(\tau ,{\text{X}}_{n})$$

where $\bar{\phi }(\tau ,X_{n})$ represents the physical tilted phase given by [16]:

(5) $$\bar{\phi }(\tau ,{\text{X}}_{n})=[2\pi h{\left[\sum_{i=0}^{n-L}u_{i}\right]}_{p}{+4\pi h\sum_{i=0}^{L-1}u_{n-i}q(\tau +iT)+W(\tau )]}_{2\pi }$$

with:

(6) $$W(\tau )=\frac{\pi h(M-1)\tau }{T}-2\pi h(M-1)\sum_{i=0}^{L-1}q(\tau +iT)+(L-1)(M-1)\pi h$$

where the operator ${\left[\cdot \right]}_{x}$ is modulo x operator, q(t) is the integral of g(t) and $q(t)=\int_{-\infty }^{T}g(t\prime )dt\prime$.

### 2.2. PSD

**Figure 2:**
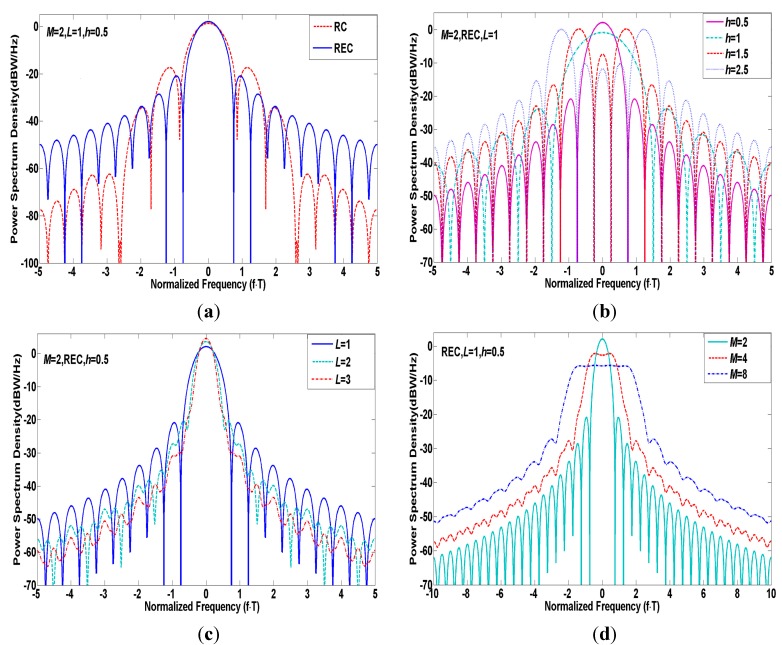
(**a**) PSD of CPM signals with different g(t); (**b**) PSD of CPM signals with different h; (**c**) PSD of CPM signals with different L; (**d**) PSD of CPM signals with different M.

As we know, PSD of modulated signals have a direct effect on tracking performance, ability of multi-path mitigation and compatibility, and the PSD of CPM is derived as follows:

If M is assumed even, the autocorrelation function of CPM can be given by [17]:

(7) $$ℜ(\tau )=\frac{1}{T}\int_{0}^{T}\prod_{k=1-L}^{\left\lfloor \tau /T\right\rfloor }\frac{1}{M}\frac{\sin2\pi hM[q(t+\tau -kT)-q(t-kT)]}{\sin2\pi h[q(t+\tau -kT)-q(t-kT)]}dt$$

where τ denotes the correlation time, and ⌊x⌋ is the maximum integer below x. According to the Wiener-Khintchine theorem, the PSD of CPM derived from Fourier transformation of ℜ(τ) is written as:

(8) $$\begin{array}{l} P(f)=2\{\int_{0}^{LT}ℜ(\tau )\cos2\pi f\tau d\tau +\frac{1-\psi (jh)\cos2\pi fT}{1+\psi^{2}(jh)-2\psi (jh)\cos2\pi fT}\cdot \int_{LT}^{(L+1)T}ℜ(\tau )\cos2\pi f\tau d\tau \\ \;-\frac{\psi (jh)\sin2\pi fT}{1+\psi^{2}(jh)-2\psi (jh)\cos2\pi fT}\cdot \int_{LT}^{(L+1)T}ℜ(\tau )\sin2\pi f\tau d\tau \} \end{array}$$

with ψ(jh)=sinMπh/Msinπh. It is noteworthy that the parameters including M, L, h and g(t) determine the spectral characteristics together, as is shown in Figure 2.

Obviously, a longer L or smoother g(t) can effectively decrease the PSD amplitude of side lobes and concentrate more energy into the main lobe while a bigger M or h would tend to extend the main lobe. It is amazing to find that the PSD of CPM behaves like BOC signals with spectrum splitting in the case of h > 1. The special subclass of CPM with tuned parameters can resemble the BOC modulation spectrum and yield comparable performance in terms of tracking accuracy, multipath mitigation, anti-jamming, and compatibility [11]. In this instance, if we can design two specific modulation schemes based on the CPM family successfully satisfying the S and C band requirements, that would reduce the complexity of the required hardware and software blocks and accelerate the practicality of multi-band combined navigation technology.

Considering the strict bandwidth restrictions for S and C bands as well as implementation complexity of CPM, this paper puts forward two specific CPM signals with M=2, h=1.5, 2RC and M=4, h=0.5, 2RC denoted as B*M*2RC and Q*M*2RC for the S and C bands, respectively, whose carrier frequencies are separately close to 2491 MHz and 5020 MHz in the S and C bands.

## 3. Evaluation Criterion

The GNSS modulation signal analytical methods are presented in this section and the tracking performance, multi-path mitigation, anti-jamming performance and compatibility are used as performance evaluation standards, which provide significant references on satellite navigation signal design.

### 3.1. Tracking Performance

Gabor bandwidth and code tracking errors are important technical indexes for evaluating the tracking performance. Based on a coherent early-minus-late (EML) code tracking loop, the code tracking errors in additive white Gaussian noise (AWGN) are defined as [18]:

(9) $${\sigma_{\varepsilon }}^{2}=\frac{B_{L}(1-0.5B_{L}T_{i})\int_{-B/2}^{B/2}G(f)\sin^{2}(\pi fd)df}{{\left(2\pi \right)}^{2}(C/N_{0})[\int_{-B/2}^{B/2}fG(f)\sin(\pi fd)df{]}^{2}}$$

where the $B_{L}$ denotes the tracking loop bandwidth, G(f) is the PSD of the signal that is normalized to unit power over infinite transmission bandwidth and symmetric to the carrier frequency, d denotes the correlation time spacing between the early and late reference signals, $C/N_{0}$ is the carrier-to-noise ratio (CNR), B is the receiver pre-filtering bandwidth and $T_{i}$ is the coherent integration time. Assuming that the signal is ideal and $B_{L}T_{i}$ is small enough, the Equation (9) in the limit defined as d is vanishingly small and becomes:

(10) $$\begin{array}{l} \sigma_{\varepsilon ,\;d\to 0}^{2}≅\sigma_{CRB}^{2}=\frac{B_{L}\int_{-B/2}^{B/2}G(f){\left(\pi fd\right)}^{2}df}{{\left(2\pi \right)}^{2}(C/N_{0})[\int_{-B/2}^{B/2}\pi f^{2}dG(f)df{]}^{2}}\\ \text{ =}\frac{B_{L}\pi^{2}d^{2}\int_{-B/2}^{B/2}f^{2}G(f)df}{{\left(2\pi \right)}^{2}(C/N_{0})\pi^{2}d^{2}[\int_{-B/2}^{B/2}f^{2}G(f)df{]}^{2}}\\ \text{ =}\frac{B_{L}}{{\left(2\pi \right)}^{2}(C/N_{0})\int_{-B/2}^{B/2}f^{2}G(f)df} \end{array}$$

with:

(11) $$\Delta f_{Gabor}=\sqrt{\int_{-B/2}^{B/2}f^{2}G(f)df}$$

where $\sigma_{CRB}^{2}$ and $\Delta f_{Gabor}$ are referred to as the Cramér-Rao lower bound and Gabor bandwidth, respectively. From Equation (10), it is obvious that the Gabor bandwidth can be approximately interpreted as Cramér-Rao lower bound and the greater the Gabor bandwidth, the better the code tracking accuracy.

### 3.2. Multi-Path Error Envelopes

The multi-path errors is one of the dominant error sources in GNSS. The multi-path error envelopes and average multi-path errors are valuable indexes to evaluate the multi-path mitigation ability. The received base-band signals disturbed by other reflected signals can be expressed as [19]:

(12) $$r(t)=a_{0}e^{j\psi_{0}}x(t-\tau_{0})+\sum_{n=1}^{N}a_{n}e^{j\psi_{n}}x(t-\tau_{n})$$

where $a_{0}$, $\tau_{0}$ and $\psi_{0}$ are the amplitude, delay and phase of the direct signal. Likewise, $a_{n}$, $\tau_{n}$ and $\psi_{n}$ are the amplitude, delay and phase of reflected signals, and N denotes the number of reflected signals. If we only consider one reflected path and use a coherent EML discriminator, the discriminator output can be described as follows [20]:

(13) $$D(\varepsilon )=a_{0}\left[ℜ(\varepsilon -\frac{d}{2})-ℜ(\varepsilon +\frac{d}{2})\right]+a_{1}\left[ℜ(\varepsilon -\Delta \tau -\frac{d}{2})-ℜ(\varepsilon -\Delta \tau +\frac{d}{2})\right]\times \cos(\Delta \psi )\equiv 0$$

where Δτ and Δψ are the delay and carrier phase difference between the multi-path and direct signals with $\Delta \tau =\tau_{1}-\tau_{0}$ and $\Delta \psi =\psi_{1}-\psi_{0}$ separately, and ε denotes the multi-path errors. To explore the theoretical lower bound of the multi-path errors, the cases Δψ=0 and Δψ=π corresponding to the worst multi-path errors are considered. By the definition of the Maclaurin series, the Equation (13) can be simplified as:

(14) D(ε)≈D(0)+D′(0)ε

According to the Wiener-Khintchine theorem, D(0) and D′(0) can be obtained by substituting ‘0’ into Equation (13) and the corresponding first-order derivative, *i.e.*,

(15) $$D(0)=\pm 2a_{1}\int_{-B/2}^{B/2}G(f)\sin(-2\pi f\Delta \tau )\sin(\pi fd)df$$

(16) $$D\prime (0)=4\pi a_{0}\int_{-B/2}^{B/2}fG(f)\sin(\pi fd)df\pm 4\pi a_{1}\int_{-B/2}^{B/2}fG(f)\cos(-2\pi f\Delta \tau )\sin(\pi fd)df$$

By combining Equations (14)–(16), the multi-path error envelopes can be estimated eventually by:

(17) $$\varepsilon (\Delta \tau )\approx \frac{\pm \tilde{a}\int_{-B/2}^{B/2}G(f)\sin(2\pi f\Delta \tau )\sin(\pi fd)df}{2\pi \int_{-B/2}^{B/2}fG(f)\sin(\pi fd)[1\pm \tilde{a}\cos(2\pi f\Delta \tau )]df}$$

with $\tilde{a}$ multi-path to direct ratio (MDR) namely $\tilde{a}=a_{1}/a_{0}$. The corresponding average multi-path errors can be given by:

(18) $$\varepsilon_{av}(\Delta \tau \prime )=\frac{1}{\Delta \tau \prime }\int_{0}^{\Delta \tau \prime }\frac{\|\varepsilon_{0}(\Delta \tau )\|+\|\varepsilon_{\pi }(\Delta \tau )\|}{2}d\Delta \tau$$

where $\varepsilon_{0}(\Delta \tau )$ and $\varepsilon_{\pi }(\Delta \tau )$ are the multi-path errors under the conditions Δψ=0 and Δψ=π.

### 3.3. Compatibility

For ensuring normal GNSS work and to realize interoperability to maximize the benefits of GNSS users, good compatibility is necessary. The spectral separation coefficient (SSC) is a fundamental measure of compatibility among GNSS signals and reflects the degree of interference imposed on a signal by other GNSS signals. The SSC is defined as the inner product of PSD between desired and interfering signals, as follows [21,22,23]:

(19) $$\chi_{s,J}=\int_{-B/2}^{B/2}G_{s}(f)G_{J}(f)df$$

where $G_{s}(f)$ and $G_{J}(f)$ are the PSD of the desired and interfering signals separately, where both of them are normalized to unit power over an infinite transmission bandwidth.

### 3.4. Anti-Jamming Performance

The narrowband-jamming and matched-spectrum-jamming are the main threats to the pseudo code and carrier tracking as well as the demodulation process. In order to effectively evaluate the anti-jamming ability of navigation signals against the above interferences, the paper introduces four parameters, including anti-narrowband-jamming merit factor $Q_{DemAJNW}$ and anti-matched-spectrum-jamming merit factor $Q_{DemAJMS}$ for the demodulation process, and the anti-narrowband-jamming merit factors $Q_{CTAJNW}$ and anti-matched-spectrum-jamming merit factor $Q_{CTAJMS}$ for the code tracking process.

The demodulation performance mainly lies in the coding properties and effective signal-to-noise ratio $\left(E_{b}/N_{0}\right)$ of received signals. When there exists noise and one non-white interfering signal with high power, the effective $\left(E_{b}/N_{0}\right)$ is approximately expressed by:

(20) $${\left(E_{b}/N_{0}\right)}_{eff}=\frac{C}{R(N_{0}+J\chi_{s,J})}\approx \frac{C}{J}\frac{1}{R\chi_{s,J}}\propto \frac{1}{R\chi_{s,J}}$$

where R denotes the message rate and J is the received power of the interfering signal. Neglecting the constant term of C/J, a quantity called anti-jamming merit factor for the demodulation process can be obtained by converting Equation (20) into a dB level version, *i.e.*:

(21) $$Q_{DemAJ}=10\times \log_{10}(\frac{1}{R\chi_{s,J}})[\mathrm{dB}]$$

As for narrowband-jamming, we take a delta function to describe the PSD of the interfering signal as:

(22) $$G_{J}(f)=\delta (f-f_{J})$$

where $f_{J}$ is the carrier frequency offset. Through Equations (19), (21) and (22), we find that the demodulation performance would be deteriorated to a great extent when $f_{J}$ corresponds to the peak of the desired signal PSD. Thus Equation (21) can be further modified as follows:

(23) $$Q_{DemAJNW}=10\times \log_{10}\left[\frac{1}{R\times \max[G_{s}(f)]}\right][\mathrm{dB}]$$

Similarly, when $G_{J}(f)$ is equal to $G_{s}(f)$, the lower bound of matched-spectrum-jamming in the demodulation process can be given approximately by:

(24) $$Q_{DemAJMS}=10\times \log_{10}\left[\frac{1}{R\times \int_{-B/2}^{B/2}{G_{s}}^{2}(f)df}\right][\mathrm{dB}]$$

In terms of coherent EML code tracking loop, the effective $C/N_{0}$ is defined by [7,24]:

(25) $${\left(\frac{C}{N_{0}}\right)}_{eff}=\frac{C}{N_{0}+J\eta_{s,J}}$$

with code tracking spectral sensitivity coefficient $\eta_{s,J}$ as:

(26) $$\eta_{s,J}=\frac{\int_{-B/2}^{B/2}G_{s}(f)G_{J}(f)\sin^{2}(\pi fd)df}{\int_{-B/2}^{B/2}G_{s}(f)\sin^{2}(\pi fd)df}$$

If it is assumed that the interfering power is high enough and d is relatively small, then Equation (26) becomes:

(27) $$\underset{d\to 0}{\lim}{\left(\frac{C}{N_{0}}\right)}_{eff}\approx \frac{C}{J}\frac{1}{\eta_{s,J}}=\frac{C}{J}\frac{\int_{-B/2}^{B/2}G_{s}(f)f^{2}df}{\int_{-B/2}^{B/2}G_{s}(f)G_{J}(f)f^{2}df}\propto \frac{\int_{-B/2}^{B/2}G_{s}(f)f^{2}df}{\int_{-B/2}^{B/2}G_{s}(f)G_{J}(f)f^{2}df}$$

Thus based on Equation (27), anti-jamming for code tracking process can be expressed in dB as:

(28) $$Q_{CTAJ}=10\log_{10}\left(\frac{\int_{-B/2}^{B/2}G_{s}(f)f^{2}df}{\int_{-B/2}^{B/2}G_{s}(f)G_{J}(f)f^{2}df}\right)[\mathrm{dB}]$$

Like the analysis of the demodulation process above, by substituting $G_{J}(f)=\delta (f-f_{J})$ and $G_{J}(f)=G_{s}(f)$ into Equation (28), the anti-narrowband-jamming merit factors and anti-matched-spectrum-jamming merit factors for the code tracking process can be derived separately as follows:

(29) $$Q_{CTAJNW}=10\times \log_{10}\left[\frac{\int_{-B/2}^{B/2}f^{2}G_{s}(f)df}{\max[f^{2}G_{s}(f)]}\right][\mathrm{dB}]$$

(30) $$Q_{CTAJMS}=10\times \log_{10}\left[\frac{\int_{-B/2}^{B/2}f^{2}G_{s}(f)df}{\int_{-B/2}^{B/2}f^{2}G_{s}^{2}(f)df}\right][\mathrm{dB}]$$

## 4. Performance Evaluation for S Band Signals

### 4.1. PSD of S Band Signals

**Figure 3:**
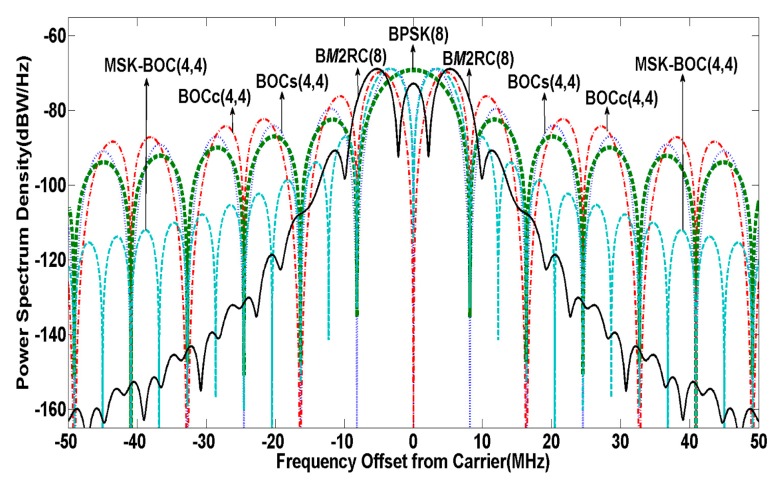
PSD of candidate signals in S band.

The cos-phase BOCc(4,4), sin-phase BOCs(4,4), MSK-BOC(4,4) [25] and BPSK(8) have been presented as candidates for the future Compass system in the S band [26]. The normalized (unit power over infinite transmission bandwidth) PSD of the above signals as well as B*M*2RC(8) are shown in Figure 3. Compared with other candidates, the proposed B*M*2RC(8) reveals a stronger spectrum roll-off in the side lobes, and performs even 35 dB lower than MSK-BOC(4,4) at ±30 MHz while still maintaining the characteristic of spectrum splitting.

### 4.2. Performance Analysis

**Figure 4:**
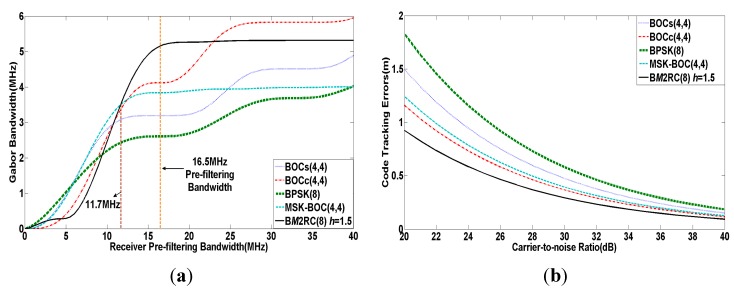
(**a**) Gabor bandwidth of modulation candidates for S band; (**b**) Code tracking errors of modulation candidates for S band.

Figure 4 shows the Gabor bandwidth and code tracking error curves of the above signals under the assumption that the receiver pre-filtering bandwidth B is equal to 16.5 MHz, tracking loop bandwidth $B_{L}$ is 1Hz and the correlation time spacing between the early and late reference signals d is 0.1 chip. As we see from Figure 4a, the Gabor bandwidth of B*M*2RC(8) is approaching its maximum at around 16.5 MHz and greater than other modulations when B approximately exceeds 11.7 MHz.

Meanwhile, the B*M*2RC(8) gives the minimum code tracking error among the analyzed signals as seen in Figure 4b. Therefore, the B*M*2RC(8) indicates a better code tracking performance than other candidates for 16.5 MHz of S band.

**Figure 5:**
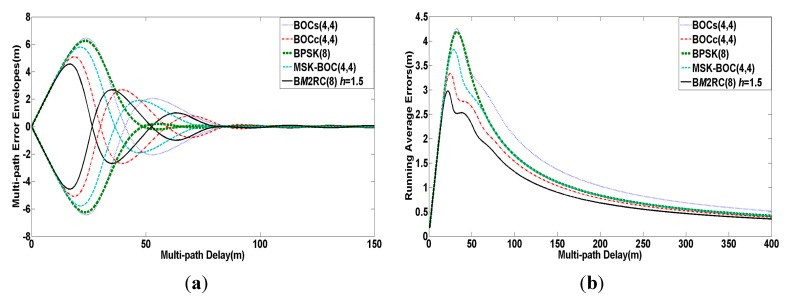
(**a**) Multi-path error envelopes of modulation candidates for S band; (**b**) Average multi-path errors of modulation candidates for S band.

An analysis of multi-path resistance performance for S band signals is estimated by multi-path error envelopes and running average multi-path errors in Figure 5, where MDR $\tilde{a}$ is fixed at −6 dB and all other parameters are the same as the previous simulation. As seen in Figure 5, the minimum quantity of running average errors can be provided by B*M*2RC(8) when the multi-path delay is changed from 0 to 400 m, while it maintains the lowest multi-path error envelope amplitude when the multi-path delay is in the range of [0, 25 m]. We are able to deduce that B*M*2RC(8) achieves the best multi-path mitigation performance among the above modulations as a result of cutting down the side lobe amplitude to a large extent.

The anti-jamming performance in terms of $Q_{DemAJNW}$ and $Q_{DemAJMS}$ for the demodulation process, and $Q_{CTAJNW}$ and $Q_{CTAJMS}$ for the code tracking process are calculated in Table 1, where B and R are set as 16.5 MHz and 50 bps, respectively. Note that the differences among various signals are very slight and even less than 1.3 dB in each anti-jamming merit factor, thus they have similar or comparable anti-jamming ability performance.

**Table 1:** Anti-jamming merit factors of modulation candidates in S band [dB].

Merit Factors	B*M*2RC(8)	BOCs(4,4)	BOCc(4,4)	BPSK(8)	MSK-BOC(4,4)
$Q_{CTAJMS}$	70.2910	70.7160	71.0772	72.8167	70.5057
$Q_{CTAJNW}$	68.0721	67.8803	67.8264	69.1297	68.3023
$Q_{DemAJMS}$	53.5508	53.9370	55.3525	53.9137	53.1460
$Q_{DemAJNW}$	51.8673	51.9276	52.6869	52.1400	51.7845

BPSK(1), BPSK(4), BPSK(8) and BOCc(1,1) have been investigated as possible signals in the S band for the Galileo system centered on a carrier frequency close to 2491 MHz with a 16.5 MHz receiver pre-filter [3,27]. The compatibility between the candidates in the future Compass system, including B*M*2RC(8) and the Galileo candidates is shown in Table 2. We can observe that the spectral separation degree of B*M*2RC(8) with BOCc(1,1), BPSK(4), and BPSK(8) is remarkably superior to the others, except for BOCc(4,4), because it contains less low frequency components within the receiver bandwidth that is likely to result in relatively low spectrum efficiency or high spectrum leakage. The compatibility between B*M*2RC(8) and BPSK(1) is a bit worse than some of the candidates, yet it is still acceptable.

**Table 2:** SSC of modulation candidates between Compass and Galileo in the S band [dB].

SSC	B*M*2RC(8)	BOCs(4,4)	BOCc(4,4)	BPSK(8)	MSK-BOC(4,4)
BOCc(1,1)	−75.7768	−72.9434	−79.0989	−70.1492	−73.3354
BPSK(1)	−73.2353	−79.9343	−86.0651	−69.3153	−80.3243
BPSK(4)	−74.9872	−73.9122	−80.0727	−69.9253	−74.3046
BPSK(8)	−74.3592	−72.1678	−75.2494	−70.9034	−72.0851

## 5. Performance Evaluation for C Band Signals

### 5.1. PSD of C Band Signals

The ITU clearly stipulates that the future C band signals can’t interfere with the normal radio astronomy services (RA, 4990–5000 MHz) and microwave landing system (MLS, 5030–5150 MHz) [4,13]. To meet the strict compatibility condition constraints of the C band, the PSD of modulated signals should have the following characteristics: (1) the majority of power should be concentrated into the main lobe; (2) a stronger spectrum roll-off in side lobes should be provided in order to significantly cut down spectrum leakage or interference to RA and MLS.

**Figure 6:**
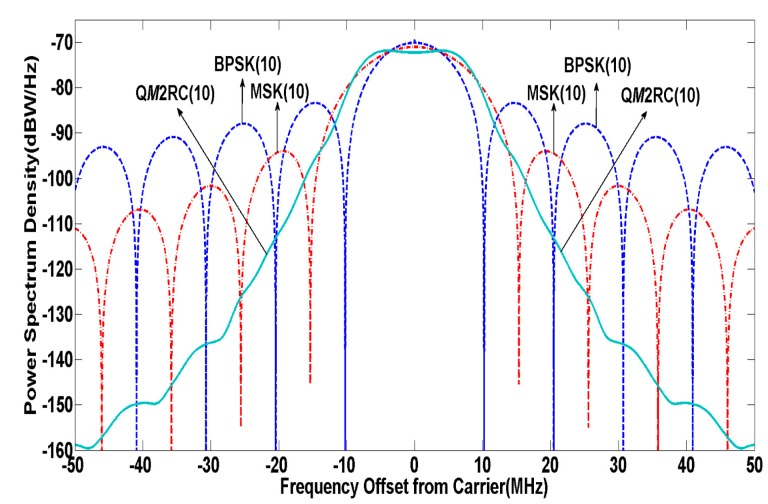
PSD of modulation candidates in C band.

To make a fair analysis, this paper compares BPSK(10), a Galileo C band candidate (MSK(10)) [13] and Q*M*2RC(10) with similar spectral occupancy within the 20 MHz bandwidth of the C band. The normalized PSD of such signals are shown in Figure 6.

It is shown that the proposed Q*M*2RC(10) can centralize most of power into the main lobe while effectively inhibiting the re-growth of spectrum side lobes compared to other competitors. In view of the stringent compatibility requirements for RA and MLS, without strong output filtering, it is impossible to limit the emissions for any signal to the level required for RA and MLS [4]. Thus, from the C band signal design point of view, the main factor to be addressed is to limit the out-of-band (OOB) emission power based on the fact that a signal with relatively low OOB emission power could significantly mitigate signal distortion caused by non-ideal filter characteristics and degrade noise level to meet strict RA and MLS constraints while also contributing to reducing design complexity and cost of filters on satellites. Table 3 assesses and compares the out-of-band loss within the 20 MHz bandwidth for C band navigation and the OOB emission power of proposed C band candidate signals at RA and MLS services, where a quantity called out-of-band loss is defined as:

(31) $$\lambda_{loss}=\left|10\times \log_{10}\left(\int_{-B/2}^{B/2}G_{s}(f)df\right)\right|$$

**Table 3:** The OOB emission power and out-of-band loss of modulation candidates in the C band.

Signal	OOB Emission Power in 4990–5000 MHz band (RA) (dBc)	OOB Emission Power in 5030–5150 MHz Band (MLS) (dBc)	Out-of-Band Loss in 5010–5030 MHz for C Band Navigation (dB)
Q*M*2RC(10)	−49.9821	−20.4880	0.0783
BPSK(10)	−20.8451	−13.5014	0.4428
MSK(10)	−29.9547	−17.6705	0.1512

As shown in Table 3, the out-of-band losses for the Q*M*2RC(10) signal within the 20 MHz bandwidth are separately 0.3645 dB and 0.0729 dB lower with respect to BPSK(10) and MSK(10). Besides, the OOB emission power of Q*M*2RC(10) in the RA and MLS band are −49.9821 dBc and −20.4880 dBc, which are even 20.0274 dBc and 2.8175 dBc lower than those of the MSK signal. Due to the high spectral efficiency and relatively low OOB emission power of the Q*M*2RC signal, the spectral separation of C band signals with RA and MLS services can be improved significantly.

### 5.2. Performance Analysis

**Figure 7:**
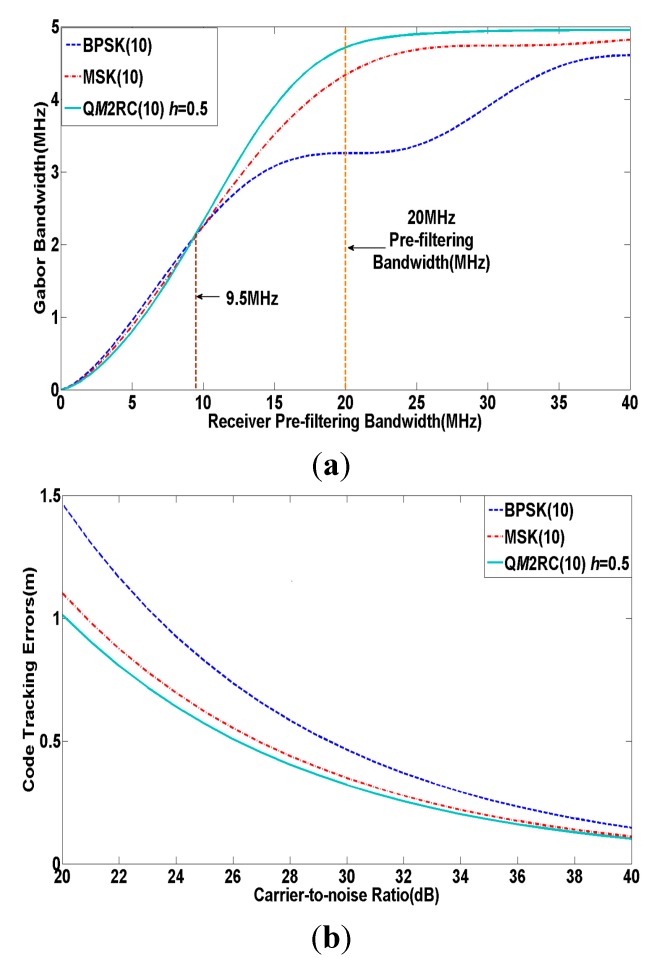
(**a**) Gabor bandwidth of modulation candidates for C band; (**b**) Code tracking errors of modulation candidates for C band.

The Gabor bandwidth and code tracking errors of the abovementioned signals are shown in Figure 7, where B is 20 MHz. It is obvious that the Gabor bandwidth of Q*M*2RC(10) is very close to that of the others below 9.5 MHz pre-filtering bandwidth, while the Gabor bandwidth of Q*M*2RC(10) appears to outperform the others and finally tends to converge to a value when B is higher than 9.5 MHz. In addition, it is also observed from Figure 7b that the proposed Q*M*2RC(10) signal can offer superior tracking accuracy performance compared to other candidates at around 20 MHz for C band navigation service.

**Figure 8:**
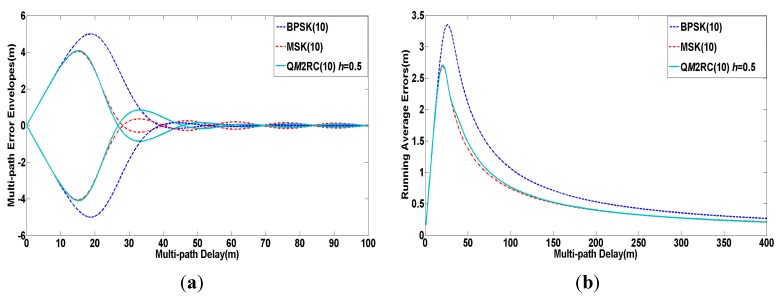
(**a**) Multi-path error envelopes of modulation candidates for C band; (**b**) Average multi-path errors of modulation candidates for C band.

Figure 8 shows the comparison of multi-path mitigation for the three candidate signals in the C band with the same parameters as the former simulation. As seen from the Figure 8, the Q*M*2RC(10) signal has almost equivalent performance to MSK(10) in the aspects of multi-path error envelopes and running average errors, and both of them are superior to BPSK(10).

Table 4 lists the anti-jamming merit factors of C band signals with 20 MHz pre-filtering bandwidth and 50 bps message rate. In the $Q_{CTAJNW}$ and $Q_{CTAJMS}$ aspects the Q*M*2RC(10) signal only behaves approximately 0.8 dB worse than MSK(10), while it reveals the best $Q_{DemAJMS}$ and $Q_{DemAJNW}$ performance. As a whole, the Q*M*2RC(10) almost shows similar or comparable anti-jamming performance as other candidates.

**Table 4:** Anti-jamming merit factors of candidate signals in the C band [dB].

Merit Factors	Q*M*2RC(10)	BPSK(10)	MSK(10)
$Q_{CTAJMS}$	73.4678	73.7855	74.2665
$Q_{CTAJNW}$	70.2342	70.0984	70.9042
$Q_{DemAJMS}$	55.5892	54.8792	55.4381
$Q_{DemAJNW}$	54.8397	52.1400	54.0212

It is assumed that the BPSK(10), MSK(10) and Q*M*2RC(10) signals centered at the same carrier frequency close to 5020 MHz are broadcast as C band signals. A receiver pre-filtering bandwidth of 20 MHz is used. Table 5 calculates the SSC of the above signals. In terms of self-SSC, the Q*M*2RC(10) is 0.15 dB and 0.71 dB lower than MSK(10) and BPSK(10), respectively, and far better than the minimum shift keying-binary coded signal (MSK-BCS) [7,28], whose self-SSC is −67.2531 dB in the C band [7]. By the above analysis, the Q*M*2RC(10) has a better compatibility performance than the others.

**Table 5:** SSC of candidate signals in the C band [dB].

SSC	Q*M*2RC(10)	BPSK(10)	MSK(10)
Q*M*2RC(10)	−72.5789	−72.6223	−72.6020
BPSK(10)	−72.6223	−71.8689	−72.2611
MSK(10)	−72.6020	−72.2611	−72.4278

## 6. Conclusions

In this paper, we introduced a universal modulation scheme based on the CPM family for multi-band combined navigation. Two specific CPM signals, namely B*M*2RC(8) and Q*M*2RC(10), are proposed as modulation solutions in the S and C bands, respectively. Theoretical analysis and simulation results show that the proposed modulation schemes can not only meet well the requirement of compatibility in the S and C bands and reduce the user terminal complexity in the multi-band combined navigation mode, but also offer better performance in the aspects of tracking accuracy, multi-path mitigation and anti-jamming compared to potential candidates such as BPSK, BOC, MSK, MSK-BOC, *etc.* Besides, the modulation schemes also provide new ideas and feasibility demonstration for signal design of multi-band combined navigation satellite systems.
